# Periodontitis and Inflammatory Bowel Disease: A Review

**DOI:** 10.7759/cureus.54584

**Published:** 2024-02-20

**Authors:** Faris I Ozayzan, Amal A Albishri, Abdulaziz E Dallak, Ahmad S Al-Qahtani, Marwa Y Mushtaq, Osamh E Dallak, Abdulaziz M Altalhi

**Affiliations:** 1 Dentistry, Ministry of Health, Jazan, SAU; 2 Dentistry, Ash Shubaykiyah Primary Health Care, Qassim, SAU; 3 Dentistry, Baish General Hospital, Jazan, SAU; 4 Dentistry, Ministry of Health, Riyadh, SAU; 5 Dentistry, Ministry of Health, Baljurashi, SAU; 6 Dentistry, Jazan University, Jazan, SAU

**Keywords:** periodontal treatment, periodontal disease and inflammatory bowel, crohn`s disease, inflammatory bowel disease, periodontitis

## Abstract

The complex relationship between periodontitis (PD) and inflammatory bowel disease (IBD) has received significant attention in recent studies. Emerging evidence suggests that the oral-gut axis plays a pivotal role in their interaction. This review provides a comprehensive, up-to-date analysis of original research from 2003 to 2023 on the PD-IBD relationship and aims to be a reference for future research. Relevant literature was sourced from the PubMed database using the keywords "periodontitis" and "inflammatory bowel disease". Additionally, a manual library search and a review of bibliographies were conducted. Of the 297 articles retrieved, 27 studies were chosen for final review. Out of these, 21 studies (78%), including both in vitro and in vivo research, indicated an association between PD and IBD. While many studies confirm a bi-directional relationship, others refute it or deem it clinically irrelevant. There is a need for more accessible studies, such as randomized trials, which also investigate the factors that could influence the outcomes to clarify the exact molecular mechanisms and clinical implications of this complex relationship.

## Introduction and background

Periodontitis (PD) is a chronic inflammatory condition that affects the periodontium, the structure surrounding the teeth [1]. Microbial dysbiosis plays a crucial role in the initiation and progression of PD by triggering an inflammatory response, which destroys the connective tissue of the gingiva, periodontal ligament, and alveolar bone [2]. Interactions between the microbiome and host inflammatory responses reinforce this dysbiosis and create a cycle of tissue damage and immune activation [3]. Clinically, PD is characterized by probing pocket depth (PPD) ≥4 mm, bleeding on probing (BOP), clinical attachment loss (CAL), radiographic evidence of alveolar bone loss (ABL), pathologic mobility, and pathologic migration of the teeth [4].

Inflammatory bowel disease (IBD) is a term that encompasses chronic inflammatory conditions of the gastrointestinal tract, and it most commonly presents as Crohn's disease (CD) and ulcerative colitis (UC) [5]. CD is characterized by transmural inflammation that can affect any part of the gastrointestinal tract. Its multifaceted etiology, involving a complex interplay between genetic predisposition and environmental factors, further contributes to its increasing prevalence [6]. Clinically, CD manifests with a range of symptoms, which include abdominal pain, diarrhea, weight loss, and fatigue [7].

UC is characterized by inflammation of the colon and rectum [8]. It is distinguished by its relapsing and remitting nature, with mucosal inflammation typically starting distally and potentially extending throughout the colon [9]. The etiology of UC remains unknown, contributing to its classification as an IBD [10]. UC presents with symptoms that show involvement of the colonic mucosa, such as bloody stools and diarrhea [11].

Emerging evidence suggests that the oral-gut axis plays a pivotal role in the interconnection between PD and IBD, which is influenced by immune mechanisms [12,13]. The ectopic colonization of the gut by oral pathogens contributes to the progression of IBD, thus establishing a link between the oral microbiome and gut inflammation [14]. Pro-inflammatory cytokines, a key element in the immune response, are implicated in the crosstalk between periodontal tissues and the gut, highlighting the systemic impact of PD. Both PD and IBD are characterized by chronic inflammation mediated by a complex interplay between genetic predisposition, environmental factors, and dysbiosis [15]. The impact of PD on IBD disease activity suggests a potential synergistic effect, where the inflammatory milieu in the oral cavity influences the systemic inflammatory burden associated with IBD [16]. Furthermore, the heightened inflammatory state in IBD patients may have systemic effects that exacerbate periodontal inflammation, impact the progression of PD, and worsen its severity. The findings from this review could significantly impact clinical practice, prompting healthcare professionals to adopt a multidisciplinary approach to diagnosis and treatment. This integrated perspective acknowledges the connection between periodontal and gastrointestinal health, potentially leading to earlier detection and more comprehensive management strategies for patients with periodontitis and inflammatory bowel disease.

This article aims to provide a comprehensive review of recent studies that have assessed the associations between PD and IBD and to act as a reference for future studies. We also highlight gaps and limitations in the current understanding of the PD-IBD bidirectional relationship and provide recommendations for future research.

## Review

Methods

The primary objective of this review was to elucidate the relationship between periodontitis (PD) and inflammatory bowel disease (IBD). Independent searches were conducted by two reviewers in the PubMed database using descriptors and - Medical Subject Heading (MeSH) terms: (periodontitis) AND (inflammatory bowel disease) AND (oral-systemic health). A 20-year publication window (2003-2023) was applied.

Included in the Review

The review included a diverse array of study types, such as prospective and retrospective studies, case series, and case reports, aiming to capture a broad spectrum of research on the PD and IBD relationship, and only articles published in English and available in full-text format were considered.

Excluded from the Review

Exclusions were made for literature reviews, systematic reviews, and editorials to focus on original research. Studies not published in English or those not relevant to the specified outcomes were also excluded.

**Figure 1:**
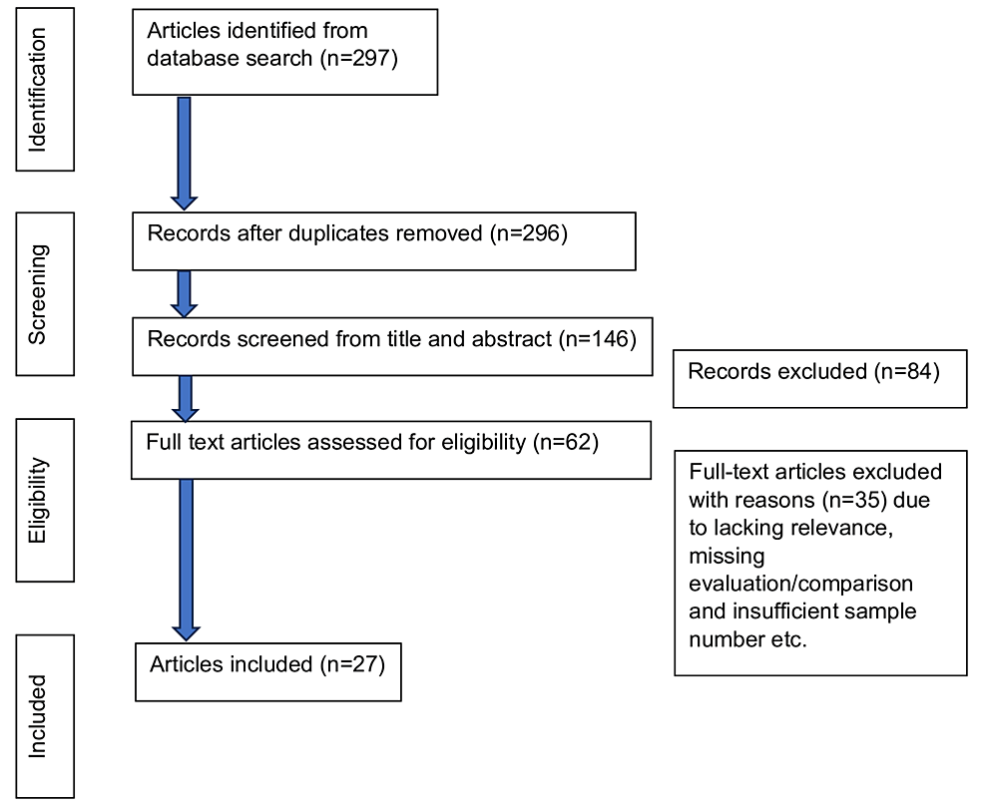
Flowchart summarizing the bibliographic search and the selection of articles

From an initial pool of 297 potentially relevant records identified, one duplicate was removed, leaving 296 articles. After screening titles and abstracts, 146 studies were excluded for not meeting the selection criteria. A detailed review of the remaining 62 studies resulted in 27 studies meeting all inclusion criteria for the final qualitative analysis. Additionally, a manual library search and review of bibliographies were performed to ensure comprehensiveness. Figure 1 provides a summary of the search and selection process. Our methodology was meticulously designed to exclude any form of bias, ensuring a neutral and comprehensive aggregation of literature on the topic.

Discussion

In Vivo and In Vitro Studies Supporting the Association Between Periodontal Disease and Inflammatory Bowel Disease

The studies included in this literature review varied in criteria, design, and evaluation methods. Numerous factors, such as study design and sample size, were taken into account before drawing conclusions. All included studies were either cohort or case-control studies. A key consideration was the adjustment for confounding factors: the majority of studies attempted to control for common risk factors, such as alcohol, tobacco, and other effect modifiers. This allowed for a more even comparison between studies. After initially screening 297 articles, 27 studies were selected for final inclusion in the review. Of these, 21 out of 27 articles (78%) from both in vitro and in vivo studies indicated an association between PD and IBD.

Furthermore, 10 out of 27 studies (37%), comprising in vitro and animal research, demonstrated a link between PD and IBD and explored the molecular mechanisms of this bidirectional relationship. For instance, a study by de Mello-Neto [17] found a significant overexpression of Th1- and Th2-related cytokines in the colon of Wistar rats (p<0.05). The authors noted a significantly higher intestinal expression of proinflammatory cytokines in cases of induced PD (p<0.05), which could elucidate the mechanism behind the development of IBD in patients with PD. Another study [18] demonstrated the occurrence of PD in a mouse model of progressive CD-like ileitis. It suggested that PD and CD may have certain pathogenic mechanisms in common, such as abnormal immune responses and dysbiosis. Xiong et al. [19] utilized bioinformatics to investigate the interactions between genes and molecular pathways in PD and IBD, identifying 43 shared genes that contribute to disease progression through immune and inflammatory pathways. Their study revealed that immune cells were expressed in both PD and IBD samples, with shared genes primarily involved in immune and inflammatory processes.

Furthermore, accelerated alveolar bone loss (ABL) spontaneously occurred in mice and correlated more significantly with intestinal inflammation than periodontal inflammation. This suggests that immunopathological changes might be the underlying cause of abnormal alveolar bone metabolism [20]. These conclusions align with the findings of Zahn et al. [21], who suggested that in a mouse model of IBD, immunopathological changes could potentially cause abnormal alveolar bone metabolism. Collectively, these studies provide evidence of the molecular mechanisms underlying the shared pathophysiology of PD and IBD. The animal and in vitro studies included in the review also shed light on the role of microbiota in the pathogenesis of PD and IBD. Sohn et al. [22] discovered that the periodontal pathogen Porphyromonas gingivalis (P. gingivalis or Pg) disrupted the gut microbiota in mice after cecal transplantation, leading to intestinal inflammation. A 2022 study [23] administered salivary microbiota from healthy or PD-affected human donors to mice via gavage, and the findings demonstrated that PD contributes to the pathogenesis of colitis. In an analysis of taxonomic assignment files [24], the abundance of Porphyromonadaceae in fecal samples differed between participants with CD and the controls. After the intrarectal implantation of P. gingivalis, the disease activity index score, colonic epithelial loss, and inflammatory cell infiltration were increased. This revealed the importance of Pg in the exacerbation of IBD. The abundance of Porphyromonadaceae in fecal samples was found to differ significantly between participants with Crohn's disease and the control group. Following the intrarectal implantation of P. gingivalis, there was an increase in the disease activity index score, colonic epithelial loss, and inflammatory cell infiltration. This highlighted the significant role of P. gingivalis in exacerbating IBD. Moreover, Lin [25] observed that in mice, another periodontal pathogen, Fusobacterium nucleatum (F. nucleatum), promoted gut inflammation, epithelial barrier dysfunction, microbiota dysbiosis, and dysmetabolism, thereby aggravating UC. Collectively, these studies affirm that pathogenic periodontal microorganisms have the potential to cause human inflammatory bowel disease.

Table 1 presents a summary of the studies supporting the association between periodontal disease and inflammatory bowel disease.

**Table 1:** In vivo and in vitro studies supporting the association between periodontal disease and inflammatory bowel disease PD - periodontitis; IBD - inflammatory bowel disease; CD - Crohn's disease; UC - ulcerative colitis; IL - interleukin; TNFα - tumor necrosis factor α; NF-κB - nuclear factor kappa B

Author(s)/ Year	Type of study	Sample size	Results	Conclusions
de Mello-Neto, et al. 2023 [17]	Case-control	The study included 13 Wistar rats, with seven undergoing ligature-induced PD and six serving as controls.	In the induced PD group, intestinal tissues showed moderate infiltration of primarily mononucleated inflammatory cells, indicating a specific immune response.	Induced PD was linked to an increased expression of Th1/Th2-related cytokines in the colons of Wistar rats.
Pietropaoli, et al. 2014 [18]	Case-control	The study involved 40 mice in total, with 20 infected mice and 20 serving as control mice.	In the control mice, the severity of PD was not as pronounced. Notably, a strong positive correlation was observed between the severity of ileitis and alveolar bone loss in infected mice, regardless of age.	The study showcased the development of PD in a mouse model simulating progressive CD-like ileitis.
Xiong, et al. 2023 [19]	Cohort	The study included 648 cases of PD and 182 controls.	The study pinpointed 43 upregulated genes in common between periodontal disease and inflammatory bowel disease by intersecting the differentially expressed genes in both conditions.	The study investigated the potential association between PD and IBD through transcriptomic analysis, identifying the IGKC and COL4A1 genes as characteristic markers.
Qiao, et al. 2022 [20]	Case-control	The study utilized IL-10 knockout (IL-10–/–) and age-matched wild-type male mice.	Correlation analysis further showed that in IL-10(-/-) mice, alveolar bone loss was positively correlated with the extent of intestinal inflammation.	Immunopathological alterations could potentially be the underlying cause of abnormal alveolar bone metabolism.
Zhan, et al. 2023 [21]	Experimental	Differential gene expression analysis and weighted gene co-expression network analysis were conducted on PD and Crohn's disease samples.	The enrichment analysis revealed that the identified genes were predominantly involved in IL-10 signaling and the inflammatory response. Further, immune infiltration analysis highlighted a variation in the proportion of neutrophils in cases of PD and CD compared to control groups. This was determined by scoring neutrophils based on their expression of a PD-related gene set within the single-cell RNA sequencing (scRNA-seq) dataset of IBD. The enrichment analysis also indicated that in the high-score group, pathways such as inflammatory response, TNFα signaling via NF-κB, and interferon-gamma response were upregulated. This group exhibited a higher expression of pro-inflammatory cytokines and chemokines compared to the low-score group.	This study uncovers a previously unidentified mechanism connecting PD and inflammatory bowel disease IBD through the interaction of crosstalk genes and neutrophils, laying a theoretical foundation for future research in this area.
Sohn, et al. 2022 [22]	Case-control	The study included twelve wild-type mice, divided equally, with six as cases and six as controls.	Oral administration of Porphyromonas gingivalis (Pg) resulted in ileal inflammation and changes in gut microbiota, characterized by a decrease in bacterial diversity. This occurred despite the absence of Pg in the lower gastrointestinal tract.	This study is the first to elucidate the mechanism behind Porphyromonas gingivalis (Pg)-mediated intestinal inflammation. It demonstrates that Pg indirectly induces intestinal IL9+ CD4+ T cells and inflammation by altering the gut microbiota.
Qian, et al. 2022 [23]	Case-control	Between 18 and 20 mice received salivary microbiota from either healthy donors or those with PD, as well as saline.	In dextran sulfate sodium (DSS)-induced colitis, the administration of salivary microbiota from periodontal disease altered the levels of IBD-associated microbiota, including Blautia, Helicobacter, and Ruminococcus.	Based on the study findings, PD plays a role in the development of colitis by facilitating the ingestion of salivary microbiota. This confirms the systemic impact of PD and offers a new perspective on the etiology of gastrointestinal inflammatory diseases.
Lee, et al. 2022 [24]	Case-control	The study comprised four groups: a control group (n=3), a Porphyromonas gingivalis only group (n=3), a 4% dextran sulfate sodium group (n=3), and a combined 4% dextran sulfate sodium + Porphyromonas gingivalis group (n=3).	Following the intrarectal implantation of Porphyromonas gingivalis, there was a marked increase in the disease activity index score, colonic epithelial loss, and infiltration of inflammatory cells. Additionally, the levels of tumor necrosis factor-α and interleukin-6 were significantly elevated in the colons infected with Porphyromonas gingivalis.	This finding underscores the significant role of Porphyromonas gingivalis in exacerbating inflammatory bowel disease, suggesting that limiting its entry into the intestine could help in preventing the aggravation of the disorder.
Lin, et al. 2023 [25]	Case-control	Forty specific-pathogen-free mice (n=40) were randomly assigned into four groups, each consisting of 10 mice.	Alveolar bone loss, the significant presence of Fusobacterium nucleatum in the colon, changes in colon length, histopathological assessments, and the detection of inflammatory^6^cytokines changes were observed in the study (increased IL-1β and TNF-α, decreased IL-10).	Fusobacterium nucleatum contributes to the exacerbation of UC by promoting gut inflammation, impairing epithelial barrier function, disrupting microbiota balance, and causing metabolic dysfunction.

Human Studies Supporting Association Between Periodontal Disease and Inflammatory Bowel Disease

Out of the 27 studies included in this review, 11 studies (41%), comprising human case-control and cohort studies, support the association between PD and IBD. A case-control study [26] found that markers of gingivitis and PD were higher in IBD participants compared to controls. Specifically, in the Crohn's disease subgroup, high clinical activity (Harvey-Bradshaw index >10) correlated with an increased loss of periodontal attachment, demonstrating a dose-response relationship. Several recent studies have utilized data from the Taiwanese National Health Insurance Research Database (NHIRD). One case-control study [27] reported that individuals with PD had a 50% higher risk of developing UC. A cohort study [28] identified a hazard ratio of 1.36 for PD in CD participants compared to controls. Another cohort study [29], also using NHIRD data, found that participants with IBD had an increased risk of PD, particularly in the CD subgroup.

Furthermore, a Chinese cross-sectional study [30] observed significantly higher percentages of sites with PPD and clinical CAL in IBD participants compared to controls (p<0.001). The study concluded that IBD is a significant risk indicator for PD (odds ratio of 4.46). Collectively, the results of these studies underscore a clear bidirectional association between PD and IBD. Effect modifiers such as increased age, smoking, oral hygiene status, socio-economic status, and genetic susceptibility may play pivotal roles in shaping the interaction between PD and IBD. The studies included in this review have analyzed these potential modifiers to obtain nuanced insights. For instance, an increase in age appears to influence the severity of PD among IBD participants. A study [31] found that IBD notably impacted the severity of PD in participants aged 50-64 years. Another case-control study [32] found that PD was more prevalent, severe, and extensive in IBD participants compared to controls, with greater prevalence observed among those younger than 36 and between 36-45 years. Additionally, another study [33] revealed that significantly more IBD participants had moderate/severe PD (85.6% vs. 65.6%, p<0.001) and severe PD (36.7% vs. 25.6%, p<0.001) than controls, particularly in the 35-50 and 51-65 age groups. This study associated increased age, IBD, and higher plaque scores with PD, while in the IBD group, factors like male gender, IBD-associated surgery, IBD duration, and localization were prominent. Positive risk indicators for IBD included PD severity and higher BOP scores, while smoking was negatively associated with UC.

Other studies have further explored the role of smoking in the PD-IBD relationship. A cohort study found that PD participants had a higher risk of UC, with increased risks among subgroups aged ≥65 years, males, alcohol drinkers, current smokers, and those with reduced physical activity. Notably, current smokers aged ≥65 years with PD had a two-fold increased risk of UC compared to non-smokers of the same age group without PD. This association between PD and the risk of developing UC but not Crohn's disease was particularly strong in current smokers aged ≥65 years, especially among males, alcohol drinkers, and those with reduced physical activity [34]. In another case-control study, a higher occurrence of PD was noted in IBD participants than in the control participants (67.6%). Among smokers, UC participants had significantly more PD with a greater median PPD compared to controls. In non-smokers, CD and UC participants showed higher PPD than controls [35]. These findings underscore smoking as a crucial effect modifier, particularly in the relationship between PD and UC.

Oral hygiene practices do not uniformly act as an effect modifier across all PD populations. A Greek case-control study [36] found that children and adolescents with IBD had significantly higher needs for community periodontal treatment (p<0.001) compared to controls, with none of the IBD participants having a healthy periodontium (0% vs. 69%). This increased need for periodontal treatment was observed despite similar oral hygiene status between the two groups, suggesting that factors other than oral hygiene may influence PD severity in IBD. Further research is necessary to explore the role of oral hygiene in the PD-IBD relationship, especially among adult populations.

This review also includes a human study that investigated the role of microbes in the pathogenesis of PD and IBD. The case-control study [37] revealed that CD participants harbored significantly higher levels of Prevotella melaninogenica, Staphylococcus aureus, Streptococcus anginosus, and Streptococcus mutans at both gingivitis and PD sites compared to UC participants and controls (p<0.05). Meanwhile, UC participants showed higher levels of S. aureus (p=0.01) and Peptostreptococcus anaerobius (p=0.05) than controls, but only in gingivitis sites. The study highlighted that even with similar clinical periodontal parameters, IBD participants harbor a greater abundance of pathogenic bacteria in inflamed subgingival sites, potentially disrupting crucial microbe-host interactions. Table 2 presents summaries of human studies that support the association.

**Table 2:** Human studies supporting the association between periodontal disease and inflammatory bowel disease PD - periodontitis; IBD - inflammatory bowel disease; CD - Crohn's disease; UC - ulcerative colitis

Author(s)	Type of study	Sample size	Risk factors and comorbidities	Results	Conclusions
Vavricka, et al. 2013 [26]	Case-control	Oral examinations were conducted on 113 participants with IBD, comprising 69 individuals with CD and 44 with UC. The control group included 113 participants.	Risk factors for periodontitis in IBD patients: systemic inflammation, immune system dysregulation, smoking, use of immunosuppressants. Comorbidities associated with periodontitis in IBD: perianal disease, proctitis.	Markers of both gingivitis and PD were observed to be higher in participants with IBD compared to controls.	IBD, particularly perianal disease in CD, is associated with an increased prevalence of PD.
Lin, et al. 2018 [27]	Case-control	The study included 27,041 participants with PD, and 108,149 participants without PD served as the control group.	Risk factors for periodontitis in IBD patients: smoking. Risk factors for IBD in periodontitis patients: microbial interactions, host immune responses.	The overall incidence of subsequent inflammatory bowel disease (IBD) was similar in both groups studied. However, after adjusting for confounding factors, an increased risk of UC was observed in the PD group.	Participants with PD had a 50% higher risk of subsequently developing UC.
Chi, et al. 2018 [28]	Case-control	The study included 6,657 CD participants and 26,628 controls.	Risk factors for periodontitis in CD patients: lower income, living in rural areas, medications (steroids), lifestyle factors. Comorbidities associated with periodontitis in CD patients: hypertension, hyperlipidemia, type 2 diabetes mellitus, heart failure, renal disease.	The hazard ratio for PD among participants with CD was 1.36, compared to controls.	The study found an elevated hazard ratio for the development of subsequent PD among participants with CD compared to matched subjects without IBD.
Yu, et al. 2018 [29]	Cohort	In the IBD cohort, 27 participants (seven with CD and 20 with UC) were identified from the catastrophic illness registry. For comparison, 108 individuals were selected as controls for the non-IBD cohort.	Risk factors for periodontitis in IBD patients: gender female IBD patients, middle to high level of socio-economic status, smoking.	After adjusting for various confounding factors, the IBD group was found to have a higher risk of developing PD compared to the non-IBD group. Specifically, the CD subgroup demonstrated a notably increased risk of PD.	The study demonstrated that participants with IBD had an increased risk of developing PD compared to the non-IBD group, with this risk being particularly pronounced in the CD subgroup.
Zhang, et al. 2020 [30]	Cross-sectional	The study involved a total of 389 participants with IBD, consisting of 265 with CD and 124 with UC. Additionally, there were 265 participants in the control group.	Risk factors for periodontitis in IBD patients: alterations in oral and gut microbiota, dietary habits, systemic inflammation, smoking, socio-economic status.	Significantly higher percentages of sites with periodontal pocket depth (PPD) ≥5 mm and CAL ≥4 mm were observed in participants CD and UC compared to controls. A fully adjusted model indicated that both CD and UC are risk indicators for PD.	Participants with inflammatory IBD in China exhibit a higher prevalence, severity, and extent of dental caries and/or PD compared to controls, indicating a need for oral health education and multidisciplinary treatment.
Abrol, et al. 2022 [31]	Cohort	The study included 80 patients diagnosed with IBD (CD and UC), as well as those diagnosed with PD.	Risk factors for periodontitis in IBD patients: older age, particularly the age group of 50–64 years, bad oral hygiene.	IBD was found to influence the severity of PD in participants aged 50–64 years, who exhibited a higher odds ratio for PD severity.	Participants with IBD in the 50–64 age group demonstrated a higher odds ratio for an increased prevalence of PD.
Habashneh, et al. 2012 [32]	Case-control	The study included 260 Jordanian adults, comprising 101 with UC, 59 with CD, and 100 without IBD.	Risk factors for periodontitis in IBD patients: dietary habits, smoking, poor oral hygiene.	The prevalence of PD was significantly higher in participants with IBD compared to those without IBD, particularly in the age groups under 36 and between 36–45 years. Specifically, participants with CD exhibited a 4.9-fold higher odds ratio of having PD, while those with UC had a seven-fold higher odds ratio compared to individuals without IBD. Additionally, the severity of PD was markedly more pronounced in participants with both CD and UC than in those without IBD.	Participants with IBD exhibited a higher prevalence, severity, and extent of PD compared to those without IBD.
Baima, et al. 2023 [33]	Case-control	The study involved 180 participants with IBD, which included 117 with CD, 60 with UC, and three with unclassified IBD, along with 180 control participants	Risk factors for periodontitis in IBD patients: age, namely the age group of 36 to 65 years, bad oral hygiene, inflammatory nature of IBD.	A significantly greater proportion of participants with IBD had moderate to severe PD (85.6% vs. 65.6%) and severe PD (36.7% vs. 25.6%) compared to controls. This difference was more pronounced in the 35–50 and 51–65 age groups. Notably, there were no significant differences between CD and UC within these findings. Additionally, IBD subjects were approximately 3.5 times more likely to have moderate to severe PD.	Significant associations were observed between inflammatory IBD and PD, which were influenced by the clinical characteristics of CD and UC.
Kang, et al. 2020 [34]	Cohort	The study involved a total of 1,092,825 participants.	Risk factors for periodontitis in IBD patients: age, namely older patients, particularly those over 65 years, are identified as having a higher risk of developing UC; sex, namely male patients more than female patients; smoking; lack of exercise.	Participants with PD showed an increased risk of developing UC, a trend not observed for CD. Importantly, current smokers aged 65 and older with PD had a 1.9-fold higher risk of UC compared to non-smoking counterparts in the same age bracket without PD.	PD was significantly associated with an increased risk of developing UC but not CD, especially among current smokers aged 65 years and older.
Brito, et al. 2008 [35]	Case-control	The study included 99 participants with CD averaging 39.0 years, 80 with UC averaging 43.3 years, and 74 controls averaging 40.3 years.	Risk factors for periodontitis in IBD patients: smoking, diet, use of immunomodulators.	Significantly more participants with UC and CD had PD compared to controls.	Participants with IBD exhibited a higher decayed, missing, and filled teeth score and a greater prevalence of PD compared to controls, with smoking identified as a significant effect modifier.
Koutsochristou, et al. 2015 [36]	Case-control	The study involved a total of 55 children in remission, all from a single outpatient IBD clinic.	Risk factors for periodontitis in IBD patients: smoking, use of immunomodulators.	The need for community periodontal treatment was significantly higher in the study group compared to controls, with none of the participants in the study group having a healthy periodontium (0% vs. 69%).	This study revealed a higher frequency of dental caries, more pronounced clinical signs of gingival inflammation, and an increased need for periodontal treatment in children and adolescents diagnosed with IBD.
Brito, et al. 2013 [37]	Case-control	The study included 30 participants with IBD, comprising 15 with CD, 15 with UC, and 15 control participants.	Risk factors for periodontitis in IBD patients: specific oral bacteria, namely the significantly higher counts of bacteria such as Parvimonas micra, Prevotella melaninogenica, Peptostreptococcus anaerobius, Staphylococcus aureus, Streptococcus anginosus, Streptococcus mitis, Streptococcus mutans, and Treponema denticola in CD patients act as risk factors for periodontal disease. Complex interaction of oral bacteria with IBD.	Multiple-comparison analysis revealed significant differences in bacterial counts between groups for B. ureolyticus, C. gracilis, P. micra, P. melaninogenica, P. anaerobius, S. aureus, S. anginosus, S. intermedius, S. mitis, S. mutans, and T. denticola. Participants with CD exhibited significantly higher levels of these bacteria than those with UC in both gingivitis and PD sites. Specifically, CD participants had higher levels of P. melaninogenica, S. aureus, S. anginosus, and S. mutans compared to controls at both gingivitis and PD sites. Meanwhile, UC participants showed elevated levels of S. aureus and P. anaerobius compared to controls, but only in gingivitis sites.	The study revealed that despite having similar clinical periodontal parameters, participants with inflammatory IBD harbor higher levels of bacteria associated with opportunistic infections in inflamed subgingival sites. This increased bacterial presence could potentially disrupt the crucial microbe-host interaction.

Human Studies Show No Correlation Between Periodontal Disease and Inflammatory Bowel Disease

The literature search identified six out of 27 studies (22%) that did not support a direct relationship between PD and IBD. In two large prospective cohort studies by Williams et al. [38], no association was observed between PD, tooth loss, and the risk of IBD. These studies also noted that the lack of association was consistent across various demographics and health factors, including sex, age, body mass index, smoking status, and nonsteroidal anti-inflammatory drug use. A separate case-control study analyzed cytokine expression in 56 biofilm samples from IBD participants. As only BOP was significantly increased (p=0.001), the study concluded that IBD participants did not exhibit clinical impairment of periodontal tissues [39].

Grössner-Schreiber et al. [40] conducted a case-control study involving 62 IBD participants and 59 controls from a dental practice. They found that the mean PPD in IBD participants was 2 mm, compared to 2.2 mm in controls. Although IBD participants had more CAL of ≥4 mm (81% vs. 64%) and 5 mm (63% vs. 46%), the study did not find significant differences in PD status. However, it did observe a higher frequency of caries in IBD participants.

Furthermore, a cohort study [41] from a community hospital in Norway involving participants with UC found that 74% had PD, but no correlation was established between PD and colon activity, the Mayo score, or the duration of UC diagnosis. These studies challenge the notion of a bidirectional association between PD and IBD. The complex and multifaceted nature of both diseases may lead to diverse clinical manifestations in different populations, thereby complicating the establishment of a direct and generalizable link between PD and IBD.

Mendelian randomization (MR) study [42] utilized genetic instruments from genome-wide association study summary statistics of European descent to investigate the association between IBD and PD and vice versa. This study found that IBD was associated with an increased risk of PD. Subtype analyses further revealed that PD was associated with Crohn's disease but not UC. The MR study provided moderate evidence supporting a bidirectional causal relationship between PD and IBD. However, the authors noted that the relationship was marginal and of limited clinical relevance. Another similar MR study did not find a significant association between these conditions. The latter study cautioned against overemphasizing the clinical relevance of such associations and highlighted the need for further research to clarify the relationship between PD and IBD [43]. These findings suggest that the association between PD and IBD may not be universally established, underscoring the need for careful examination of contributing factors that might obscure a clear correlation. In addition, variations in study designs, methodologies, and population characteristics across different research endeavors could lead to conflicting results. This variability makes it challenging to draw definitive conclusions about the relationship between PD and IBD. Table 3 provides summaries of human studies that found no evidence supporting a link between periodontal disease and inflammatory bowel disease.

**Table 3:** Studies disputing the link between periodontal disease and inflammatory bowel disease PD - periodontitis; IBD - inflammatory bowel disease; CD - Crohn's disease; UC - ulcerative colitis; BOP - bleeding on probing; TNF - tumor necrosis factor

Author(s)	Type of study	Sample size	Results	Conclusions
Williams, et al. 2023 [38]	Cohort	The study included 175 cases of CD and 209 cases of UC.	After adjusting for potential risk factors, there was no significant association between PD and the risk of CD. Similarly, the pooled analysis revealed no significant association between tooth loss and the risk of CD or UC.	In two extensive prospective cohort studies, no association was observed between PD, tooth loss, and the risk of either CD or UC.
Buchbender, et al. 2022 [39]	Case-control	The study comprised three cohorts: group 0 consisted of 30 healthy controls; group 1 included 19 participants with CD; and group 2 had seven participants with UC.	The mean values for BOP and periodontal disease PD were significantly higher in the CD group compared to controls. Additionally, the expression of interleukin-10 was significantly elevated in both the CD and UC groups. The expression of matrix metallopeptidase-7 was also notably higher in the CD group. Participants with IBD who were treated with TNF inhibitors showed a significant overexpression of interleukin-10, as did those on other immunosuppressants, compared to controls.	Since only bleeding on BOP was significantly increased, the study concluded that there was no clinical impairment of periodontal tissues in participants with IBD.
Grössner-Schreiber, et al, 2006 [40]	Case-control	The study involved 62 participants seeking treatment for IBD and 59 control participants.	The findings indicate a higher frequency of dentine caries in participants with IBD, yet the periodontal assessments revealed no significant differences in periodontal health between the cases and controls.	The results indicate a higher prevalence of dentine caries in participants with IBD, yet no significant differences in periodontal health were observed between the cases and controls.
Haugbo, et al. 2023 [41]	Cross-sectional, cohort	In total, 53 participants participated in the study.	Based on the 2017 classification, 74% of the study's participants were found to have PD. However, no correlation was established between PD and clinical activity, the Mayo score, or the duration since UC diagnosis.	There was no observed correlation between PD and UC disease indices or the duration since UC diagnosis.
Wang, et al. 2023 [42]	Case-control	The study included 12,882 cases and 21,770 controls.	In the primary causal analysis, IBD as a whole was associated with an increased risk of PD. Upon examining the subtypes, UC showed an association with PD, whereas CD did not. Conversely, the reverse analysis suggested an association between PD and IBD in general. Further subtype analysis revealed a link between PD and CD, but this association was not observed with UC.	The study offered moderate evidence supporting a bidirectional causal relationship between PD and IBD. However, the observed bidirectional increased risk was marginal and potentially of limited clinical significance.
Yu, et al. 2023 [43]	Retrospective cohort	The study included 27 participants with IBD, comprising seven with CD and 20 with UC. The control group consisted of 108 participants.	In the Mendelian randomization (MR) analysis, the primary method employed was random-effects inverse-variance weighting, while the weighted median and MR Egger regression served as complementary methods. A series of sensitivity analyses were also conducted to ensure the reliability of the findings. None of these analyses revealed a significant effect of genetically proxied IBD and its subtypes on pePD), or vice versa. The subsequent sensitivity analyses did not detect any horizontal pleiotropy or heterogeneity.	Caution is advised regarding the clinical relevance of these findings, and further studies are necessary to elucidate the relationship between PD and IBD.

Future Scope

By further investigating the complex relationship between PD and IBD, future research will embark on paths that can potentially lead to substantial clinical improvements. Future studies could delve into the potential bidirectional impact of treatment strategies and explore whether interventions targeting IBD positively influence periodontal disease outcomes and vice versa [44]. Additionally, investigations into shared etiological factors, effect modifiers, and common pathways could contribute to targeted therapeutic approaches for both conditions, potentially improving patient outcomes and quality of life. Longitudinal studies assessing the evolution of the association over time and across diverse patient populations would provide valuable insights and enable personalized healthcare strategies [45].

Research studies could also explore the mechanistic aspects of the microbial link between the two conditions through in-depth sequencing techniques, as suggested by Zhang et al. [5]. As understanding of the field advances, interdisciplinary collaboration between gastroenterologists, dentists, and microbiologists will be essential for a holistic exploration of the intricate interplay between IBD and periodontitis.

Challenges and Limitations

Conducting a comprehensive literature review on the association between PD and IBD proved challenging. Firstly, heterogeneity in study designs, methodologies, and diagnostic criteria across different research works may have introduced variability in reported findings. Secondly, the temporal nature of both conditions makes it challenging to establish a causal relationship. The bidirectional interplay between PD and IBD necessitates long-term observational studies and a cautious interpretation of causation. Mendelian randomization studies, while insightful, may still be insufficient to capture the complex, multifactorial nature of the association.

Despite the growing body of observational studies suggesting the PD-IBD link, there remains a notable dearth of randomized control trials (RCTs) establishing a causal relationship. The available studies obtained during this review comprised mainly of case-control and cohort studies, which provide valuable insights but fall short of offering definitive evidence. There is a need for more robust investigations to confirm causality. The scarcity of RCTs hinders our ability to establish the precise nature of the relationship and thus develop targeted interventions. To advance our understanding, future research should prioritize well-designed RCTs that consider immunological traits and explore therapeutic implications for both conditions [46].

## Conclusions

An extensive body of research elucidates a multifaceted relationship between PD and IBD. The reviewed evidence favors a bidirectional association, where persons with IBD have an elevated risk of developing PD, and conversely, PD may influence the severity and activity of IBD. This intricate association involves a complex pathogenic network influenced by immunological traits, microbial dysbiosis, and inflammatory cascades.

Several studies emphasize the clinical significance of recognizing this interconnection, not only for oral health but also for the management of IBD. The shared inflammatory pathways and potential common etiological factors underscore the need for a holistic approach to patient care. However, there is also literature that refutes the existence or clinical relevance of this relationship. In light of the compelling evidence presented in this review, further research, especially RCTs, is required to enhance our understanding of the relationship between IBD and PD.
